# Probiotic toothpastes and mouthrinses: probiotic presence, viability, culturability, label accuracy, and effectiveness *in vitro*

**DOI:** 10.3389/froh.2026.1936217

**Published:** 2026-10-15

**Authors:** Mehraveh Saghi, Wannes Van Holm, Naiera Zayed, Katalina Lauwens, Kobe Teughels, Nico Boon, Kristel Bernaerts, Wim Teughels

**Affiliations:** 1 Department of Oral Health Sciences, Periodontology and Oral Microbiology, KU Leuven, Leuven, Belgium; 2 Center for Microbial Ecology and Technology (CMET), Department of Biotechnology, Faculty of Bioscience Engineering, Ghent University, Ghent, Belgium; 3 Department of Pharmaceutical Microbiology and Immunology, Menoufia University, Faculty of Pharmacy, Shibin El-Kom, Egypt; 4 Department of Chemical Engineering, Chemical and Biochemical Reactor Engineering and Safety (CREaS), KU Leuven, Leuven, Belgium; 5 Department of Restorative Dentistry, Universiti Malaya, Faculty of Dentistry, Kuala Lumpur, Malaysia; 6 Department of Periodontology, Chulalongkorn University, Faculty of Dentistry, Bangkok, Thailand

**Keywords:** lozenges, mouthrinse, oral health, probiotic, toothpaste

## Abstract

**Objective:**

Per their design, several oral hygiene products have antimicrobial properties to influence the oral microbiome. However, due to overconsumption, resistance to these antimicrobials is developing. In contrast, as a pro-microbial approach, probiotics hold promise for rebalancing the oral microbiome towards a symbiotic state. Despite the availability of probiotic oral products, uncertainties about the probiotic presence, culturability, and reliability of labels persist. This study assessed the culturability, effectiveness, and accuracy of probiotics listed in commercially available probiotic toothpastes and mouth rinses.

**Methods:**

A total of 16 probiotic toothpastes and 6 probiotic mouthrinses, available on the European and American markets, were analysed for the number of culturable (CFU; agar plating), live (viability qPCR), and total bacteria (qPCR), and if they indeed contain the probiotic species proclaimed (16S rRNA sequencing). The products with viable probiotics were further evaluated for their impact on an *in vitro* oral biofilm model. Additionally, culturable probiotic isolates from these products were tested for potential inhibitory effects against individual oral pathogens.

**Results:**

Not all of the examined products contained viable bacteria; instead, they contained ferments or inactivated probiotics. Only 2 toothpastes were found to contain culturable bacteria consistent with the probiotic species declared on their labels. Additionally, traces of DNA from actual probiotics were detected in 68% of the toothpastes and 50% of the mouthrinses. Furthermore, the tested products did not show any beneficial effect on an *in vitro* oral biofilm model. Similarly, the culturable probiotic isolates recovered from the products showed no inhibitory activity against selected oral pathogens.

**Conclusions:**

This study highlights that in contrast to probiotic lozenges, which are widely used for oral probiotic delivery, currently available probiotic toothpastes and mouthrinses may not consistently meet established probiotic criteria. These findings highlight the need to critically reassess their use in scientific research and the commercial use of the term probiotic, while recognizing that the present findings are specific to the product batches analysed and may not represent each brand as a whole.

## 1 Introduction

There are various approaches to maintain oral hygiene, and numerous products are available on the market. Some of these, such as dental floss, toothpicks, tongue scrapers, interdental brushes, and oral irrigators, assist in the mechanical removal of oral biofilms from tooth and tongue surfaces (1–3). While mechanical removal is effective in disrupting and detaching oral biofilms, it is often insufficient on its own; therefore, chemical agents are incorporated to enhance bacterial removal and biofilm disruption (4). The purpose of all these techniques is to control dental plaque, which is a complex biofilm composed of diverse microorganisms embedded in a matrix of extracellular polymeric substances (5, 6). Dental plaque forms on tooth surfaces and oral tissues and plays a central role in the development of oral diseases (7).

Therefore, a wide variety of toothpastes and mouth rinses containing different active ingredients are available on the market to support oral health and aid in the prevention or management of oral diseases such as gingivitis, periodontitis, caries, halitosis, and also conditions like tooth sensitivity and tooth discoloration (1, 8, 9). However, due to overconsumption, oral bacteria risk becoming resistant to antimicrobial properties used in oral care products (4, 10).

As an alternative to antibacterial strategies, pro-microbial approaches are being developed to modulate the oral microbiota without disrupting the balance between pathogenic and commensal species. This is important due to the fact that traditional antimicrobial agents often exhibit broad-spectrum activity, leading to collateral damage to beneficial microbes and promoting the emergence of antimicrobial resistance (11–13). In contrast, pro-microbial approaches aim to preserve the symbiotic state of the oral microbiome, in which commensals and potential pathogens coexist in balance with their host. Maintaining this homeostasis supports oral health and helps prevent the shift toward a dysbiotic state, which is characterized by an overgrowth of pathogenic bacteria and a suppression of beneficial microbes, often followed by oral diseases such as periodontitis and caries (14, 15).

One of these pro-microbial strategies is the use of probiotics, defined as “live microorganisms that, when administered in adequate amounts, confer a health benefit on the host.” (16). Probiotics have been shown to support oral health and maintain a healthy oral microbiome through various mechanisms of action (17). Related approaches include prebiotics, which are defined as “a substrate that is selectively utilized by host microorganisms conferring a health benefit” (18); postbiotics, defined as “a preparation of inanimate microorganisms and/or their components that confers a health benefit on the host” (19); and synbiotics, defined as “a mixture comprising live microorganisms and substrate(s) selectively utilized by host microorganisms that confers a health benefit on the host.” (20).

Probiotic-containing oral care products such as toothpastes and mouthrinses are increasingly available on the market. However, it remains unclear whether these products truly meet the definition of probiotics as live microorganisms and whether they contain probiotics in sufficient quantities. Furthermore, the culturability of the probiotics present in these products is unclear. Cells that are able to grow and form colonies on defined nutrient media are considered culturable, and the term “viable” refers to cells that remain metabolically or physiologically active (21). Therefore, cells that retain metabolic or physiological activity but fail to grow on standard culture media are described as viable but nonculturable (VBNC) cells (21). Additionally, previous research has shown that although lyophilized probiotics provide optimal stability and extended shelf life, their viability is often lower compared with their fresh or reactivated counterparts (22). This observation raises further questions regarding the effectiveness of probiotic-containing oral care products, such as toothpaste and mouthrinses, because, due to their aqueous nature, maintaining probiotic viability in these formulations can be challenging, and the storage of probiotics in these products remains a major concern. Moreover, their actual purpose in reducing oral pathogens is still in question, raising concerns that their use may be driven more by commercial interest than by scientific evidence. To address these concerns, the present study investigated the presence, bacterial viability, label accuracy, and *in vitro* effectiveness of commercially available probiotic toothpastes and mouthrinses in comparison to a probiotic lozenge as a control.

## 2 Methods

### 2.1 Selection of probiotic toothpastes and mouthrinses and inspection of product labels for probiotic content claims

A total of 16 probiotic toothpastes and 6 probiotic mouthrinses, available on the European and American markets, were selected for analysis. Products were included based on the explicit mention of probiotic content on their packaging or ingredient list. Detailed information on the selected products is provided in Table 1 (toothpastes) and Table 2 (mouthrinses). As a positive control, BioGaia® probiotic chewing tablets (BioGaia, Lund, Sweden), known to contain viable *Limosilactobacillus reuteri* DSM 17938 (*L. reuteri* DSM) and *Limosilactobacillus reuteri* ATCC PTA 5289 (*L. reuteri* ATCC), were included in the analysis. All product labels were carefully analyzed to determine whether probiotics were included, and if so, to identify the listed probiotic species, strain, and the form in which the probiotics were added (e.g., live, fermented, or inactivated probiotics). This information was recorded for each product to document the manufacturer's claims regarding probiotic content.

**Table 1:** Probiotic toothpaste products included in the studies.

Nr	Name	Probiotic content reported on the label
TP01	Gutology® Probiotic Toothpaste (120 mL)	*Lactobacillus acidophilus, Lactobacillus paracasei*
TP02	Hyperbiotics® Probiotic Toothpaste with Activated Charcoal	inactivated *Lactobacillus paracasei*
TP03	Luvbiotics® original toothpaste with probiotics	*Lactobacillus*
TP04	YUUU® advance probiotic and pine needle toothpaste	*Lactobacillus* ferment
TP05	Pro bident® New Generation Toothpaste with Active Probiotics	*Bacillus*
TP06	STIM® Probiotic Toothpaste (80 G)	*Bacillus coagulans*
TP07	Henry blooms® probiotic toothpaste	*Lactobacillus paracasei*
TP08	REACH Microbiome® Whitening Toothpaste, Natural Probiotic Ingredients	Bifida Ferment Extract Filtrate
TP09	OraLactin® ApaCare Pre- and Postbiotic Probiotic Toothpaste	*Lactobacillus* ferment
TP10	Biorepair®, Peribiome Gum Protection Toothpaste, with probiotics	*Bifidobacterium, Lactobacillus*
TP11	Gaia® natural probiotic toothpaste	*Lactobacillus* lysate
TP12	NANB® + (enzyme whitening balancing toothpaste)	ADP-1 probiotics
TP13	Sp-4® Toothpaste Probiotic Whitening Toothpaste Yayashi	*Lactobacillus acidus*
TP14	ELAA®.Lava Toothpaste|Probiotic Whitening Charcoal Mint	*Lactobacillus sakei, Lactobacillus reuteri, Lactobacillus salivarius, Lactobacillus brevis, Lactobacillus paracasei, Streptococcus thermophilus, Bifidobacterium bifidum, Bifidobacterium longum*
TP15	Dentist Plus® White Premium Toothpaste Xylitol and Probiotics	Lactoferrin, Lactoperoxidase
TP16	Be Well® Natural Toothpaste with probiotics	10 unidentified probiotic strains

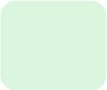
 Inactivated probiotics.

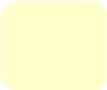
 Unknown strain.

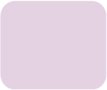
 Probiotic ferment.

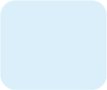
 Other components.

**Table 2:** Probiotic mouthrinse products included in the studies.

Nr	Name	Probiotic content reported on the label
MR01	Luvbiotics® Mouthwash with Oral Probiotics	*Lactobacillus*
MR02	Riven® rinse probiotic mouthwash	*Lactobacillus reuteri, Lactobacillus paracasei*
MR03	OraLactin® Pre and Postbiotic Mouthwash	*Lactobacillus* ferment
MR04	Henry blooms® on the go probiotic mouthwash	*Lactobacillus paracasei*
MR05	Truthpaste® mint probiotic mouthwash fluoride free	*Lactobacillus* ferment
MR06	Truthpaste® mint probiotic mouthwash with fluoride	*Lactobacillus* ferment

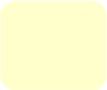
 Unknown strain.

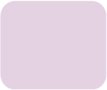
 Probiotic ferment.

### 2.2 Assessment of probiotic viability by culturing, colony-forming units counting, and species identification of culturable bacteria by Sanger sequencing

Probiotic toothpaste and mouthrinse products (Tables 1, 2) were cultured to assess the culturability of bacteria in the products. As most products contain lactobacilli, samples were plated on de Man, Rogosa, and Sharpe agar (MRS; Merck, Darmstadt, Germany) and incubated at 37° C under both anaerobic and aerobic (5% CO_2_) conditions. Products containing *Bacillus* species were additionally cultured on Luria–Bertani agar (LB; Merck, Darmstadt, Germany) at 37° C. To check for the presence of major contaminants, each product was also cultured on blood agar. Colony-forming units (CFU; dilutions with 30–300 CFU/plate) were counted to quantify culturable probiotics in each product (Table 3).

**Table 3:** Bacterial growth on MRS, blood agar, and LB: CFU and sanger sequencing analysis.

Sample	MRS agar mean CFU ± SD (log_10_)	Blood agar mean CFU ± SD (log_10_)	LB agar mean CFU ± SD (log_10_)	Sanger sequencing from MRS agar
Control	8.041 ± 0.12	NG	–	*Limosilactobacillus reuteri*
TP01	NG	NG	–	/
TP02	NG	NG	–	/
TP03	NG	NG	–	/
TP04	NG	NG	–	/
TP05	5.414 ± 0.33	6. 749 ± 0.14	6.233 ± 0.24	*Heyndrickxia coagulans*
TP06	8.238 ± 0.20	8.568 ± 0.27	8.627 ± 0.12	*Heyndrickxia coagulans*
TP07	NG	NG	–	/
TP08	NG	NG	–	/
TP09	NG	NG	–	/
TP10	NG	NG	–	/
TP11	NG	NG	–	/
TP12	NG	NG	–	/
TP13	NG	NG	–	/
TP14	NG	8.342 ± 0.44	–	*Enterococcus faecium*
	+Fungi	+Fungi		
TP15	NG	NG	–	/
TP16	NG	NG	–	/
MR01	NG	NG	–	/
MR02	NG	NG	–	/
MR03	NG	NG	–	/
MR04	NG	NG	–	/
MR05	NG	NG	–	/
MR06	NG	NG	–	/

Data are presented as mean ± SD from three biological replicates (*n* = 3).

NG: No Growth (<30 CFU/plate).

Colonies that grew on MRS agar plates after 48 h of incubation were individually isolated and re-cultured. After an additional 48 h, bacterial colonies were collected in 1 mL of sterile phosphate-buffered saline (PBS). DNA was then extracted following the protocol described by Van Holm et al. (23). The extracted DNA samples were sent to LGC Genomics GmbH, Biosearch Technologies (Berlin, Germany), for Sanger sequencing to identify the bacterial species present in the cultured samples (Table 3). Sequences were analyzed using the Basic Local Alignment Search Tool (BLAST) (24).

### 2.3 Quantification of live- and total bacterial load

Each product was diluted 1 in 10 in sterile PBS and thoroughly mixed. From these dilutions, samples were taken for DNA extraction to quantify both live and total bacterial populations using quantitative PCR (qPCR), based on the method described by Van Holm et al. (23) In brief, samples were treated with PMAxx (Biotium, Hayward, CA, USA) to inhibit amplification of DNA from dead or membrane-compromised cells, allowing specific detection of viable bacteria (v-qPCR). In parallel, untreated samples (without PMAxx) were used to measure the total cells' (both live and dead) bacterial DNA. PMAxx-treated and untreated aliquots were analysed in parallel for each sample. In addition, the BioGaia® probiotic chewing tablets (BioGaia, Lund, Sweden) were included as a control. DNA was isolated using the QIAamp® DNA Mini Kit (QIAGEN, Hilden, Germany). qPCR analysis targeted the 16S rRNA gene using universal primers (forward: P338 5′-ACTCCTACGGGAGGCAGCAG-3′; reverse: P518 5′-ATTACCGCGGCTGCTGG-3′). Based on the previously reported performance of the PMA-qPCR method, measurements below approximately 500 genome equivalents/mL (2.7 log10 Geq/mL) were considered outside the reliable range for discrimination between viable and non-viable cells and were not included in the interpretation of the results (25).

### 2.4 Identification of bacterial species and potential contaminants by sequencing

After preparing 1 in 10 dilutions from the products and extracting DNA as previously described, samples were sent for 16S rRNA gene sequencing, which was conducted by the Center for Microbial Ecology and Technology (CMET, Gent, Belgium) to identify the probiotic species present in each product as well as any potential contaminants. As sequencing controls, ZymoBIOMICS Microbial Community DNA Standards (Zymo Research Corporation, Irvine, CA, USA) were included at high (1 ng/µL) and low (0.01 ng/µL) DNA concentrations. In this sequencing run, the low-concentration mock community was amplified using three additional PCR cycles and twice the template input. The DADA2 R package was used to process the amplicon sequence data according to the pipeline tutorial as described by Callahan et al. (26) In a first quality control step, the primer sequences were removed, and reads were truncated at a quality score cut-off (truncQ = 2). Besides trimming, additional filtering was performed to eliminate reads containing any ambiguous base calls or reads with high expected errors (maxEE = 2,2). After dereplication, unique reads were further denoised using the Divisive Amplicon Denoising Algorithm (DADA) error estimation algorithm and the self-consistent sample inference algorithm (with the option pooling = TRUE). The obtained error rates were inspected, and after approval, the denoised reads were merged. Subsequently, an additional clean-up step was implemented. When the total amount of target over all samples was lower than or equal to 1, the ASV was removed. Input DNA concentrations were not normalized across samples prior to amplification. Consequently, samples with low DNA input and low sequencing depth may show a relatively greater contribution of background bacterial DNA originating from sources such as reagents, laboratory handling, or cross-contamination. This was particularly relevant for samples yielding fewer than 1,000 reads, and taxa detected at very low abundance were therefore not considered conclusive. Finally, the ASV table obtained after chimera removal was used for taxonomy assignment using the Naive Bayesian Classifier and the DADA2 formatted Silva v138.1 (27). More specifically, taxonomic assignment was performed using the DADA2 assignTaxonomy function with the SILVA nr99 v138.1 DADA2 training set (minBoot = 80, tryRC = TRUE) (27). Species-level assignment was subsequently performed using the DADA2 addSpecies function with the SILVA species assignment v138.1 database (tryRC = TRUE, allowMultiple = 3).

### 2.5 Evaluation of the effect of viable probiotic products on an *in vitro* oral biofilm model

A multispecies bacterial community (Table 4) was cultivated using a Biostat-B Twin bioreactor, following the method described by Slomka et al. (28). Biofilms were developed on hydroxyapatite discs (HADs; Hitemco Medical, New York, United States), which were placed in 24-well plates. Samples from the bioreactor were diluted 1 in 10 in fresh Brain Heart Infusion-2 broth supplemented with 10 mM glycerol (BHI-2-gly) and used to inoculate the HADs (29). The well plates were incubated under microaerophilic conditions at 37° C for 48 h with gentle shaking (170 rpm) to allow biofilm formation. After 48 h, the biofilms were brushed using a micro brush (Micro Applicator Fine Pearl Purple, Demedent B.V., Cuijk, Netherlands) and exposed to diluted 1 in 5 slurries of selected toothpaste products containing viable and culturable probiotics that matched their labels for 3 min at 250 rpm. Biofilms were incubated for an additional 24 h under the same conditions. After this period, they were detached from the HADs using 0.05% trypsin-EDTA. PBS was used as a negative control, and 0.2% chlorhexidine (CHX) as a positive control. To quantify viable cells specifically, the recovered biofilm cells were treated with PMAxx, followed by DNA extraction as described by Van Holm et al. (23).

**Table 4:** Oral bacteria used in the biofilm model.

Species	Microbial category
*Aggregatibacter actinomycetemcomitans* ATCC 43718	Periodontal pathobiont
*Prevotella intermedia* ATCC 25611	
*Porphyromonas gingivalis* ATCC 33277	
*Fusobacterium nucleatum* ATCC 20482	
*Actinomyces viscosus* DSM 43327	Anaerobic commensal
*Actinomyces naeslundii* ATCC 51655	
*Veillonella parvula* DSM 2008	
*Streptococcus mutans* ATCC 20523	Cariogenic streptococci
*Streptococcus sobrinus* ATCC 20742	
*Streptococcus oralis* DSM 20627	Commensal streptococci
*Streptococcus sanguinis* LM14657	
*Streptococcus gordonii* ATCC 49818	
*Streptococcus mitis* DSM 12643	
*Streptococcus salivarius* TOVE-R	

### 2.6 Assessment of inhibitory activity of isolated probiotics against selected oral pathogens

Based on earlier results in this study, *Bacillus coagulans* (Bc) was identified as the only viable and culturable species in two tested toothpaste products. To further evaluate its potential inhibitory effects, *in vitro* experiments were conducted using six common oral pathogens: *Fusobacterium nucleatum* (Fn), *Porphyromonas gingivalis* (Pg), *Prevotella intermedia* (Pi), *Aggregatibacter actinomycetemcomitans* (Aa), *Streptococcus mutans* (Sm), and *Streptococcus sobrinus* (Ss) (Table 4).

The pathogens Fn, Pg, and Pi were grown on blood agar while incubated under (5% CO_2_) anaerobic conditions, whereas Aa, Sm, and Ss were incubated under aerobic conditions. Each pathogen was first cultured overnight in Brain Heart Infusion (BHI) broth, while *B. coagulans* was grown separately in Man, Rogosa and Sharpe (MRS) broth anaerobically. All bacterial cultures were adjusted to a concentration of 10^8^ cells/mL using flow cytometry (FCM). Pathogens were then incubated for 6 h at 37° C in BHI-2-Cyc-gluc without mucin (modified BHI2-Cys) broth according to Van Holm et al. (23), either alone or in co-culture with *B. coagulans*, maintaining their respective aerobic or anaerobic incubation conditions.

After the 6 h incubation, the samples were centrifuged at 8,000 rpm for 5 min. Cell pellets were resuspended in 1 mL of sterile PBS. Viability was assessed through PMAxx treatment, followed by DNA extraction (as previously described) and qPCR using species-specific primers (23). Additionally, the pH of the supernatants was measured to evaluate whether environmental acidification contributed to a reduction in pathogen viability.

### 2.7 Further investigation of the potential postbiotic effect of *Bacillus coagulans*

To investigate the potential postbiotic inhibitory effects of *Bacillus coagulans* (19), different medium conditions were tested. Following a 6 h incubation of the selected oral pathogens alone or in co-culture with *B. coagulans* in modified BHI2-Cys medium, while maintaining their respective aerobic (5% CO_2_) or anaerobic conditions, the cultures were centrifuged at 5,000 × g for 5 min. The supernatants were then filter-sterilized using 0.22 µm membrane filters to obtain cell-free supernatants (CFS).

The pathogens were first cultured overnight in BHI and adjusted to 10^8^ cells/mL using FCM. Each pathogen was then incubated under the following seven conditions: (1) in *B. coagulans* CFS, (2) in pH-adjusted (pH 7.4) *B. coagulans* CFS, (3) in co-culture pathogen with *B. coagulans CFS*, (4) in co-culture pathogen with *B. coagulans* in pH-adjusted CFS, (5) in pathogens alone CFS, (6) in pH-adjusted pathogen alone CFS, and (7) in modified BHI2-Cys medium. After 6 h of incubation at 37° C under the appropriate oxygen conditions for pathogens as described earlier, all samples were analyzed by flow cytometry (FCM) to assess bacterial viability, following the protocol described by Van Holm et al. (23).

### 2.8 Statistical analysis

Statistical analysis was calculated using R 4.2.2 (https://cran.r-project.org/). Data were first tested for normality of residuals and homogeneity of variances, assessed using the Shapiro–Wilk and Levene's tests, respectively. Paired data were analysed using a paired *t*-test, while unpaired data were analysed using an independent two-sample *t*-test. Bonferroni correction was applied when multiple comparisons were performed (95% CI).

## 3 Results

### 3.1 Label-based assessment of probiotic claims in oral care products

Label analysis of the products (Tables 1, 2) showed that 3 toothpastes (YUUU® advance probiotic and pine needle toothpaste, REACH Microbiome® Whitening Toothpaste, OraLactin® ApaCare Pre- and Postbiotic Probiotic Toothpaste) and 3 mouth rinse products (OraLactin® Pre and Postbiotic Mouthwash, Truthpaste® mint probiotic mouthwash fluoride free, Truthpaste® mint probiotic mouthwash with fluoride) claimed to use probiotic ferments. Additionally, 2 toothpaste products [Hyperbiotics® Probiotic Toothpaste with Activated Charcoal, NANB® + (enzyme whitening balancing toothpaste)] list inactivated probiotics. Three toothpastes (Luvbiotics® original toothpaste with probiotics, Pro bident® New Generation Toothpaste with Active Probiotics, and Be Well® Natural Toothpaste with probiotics) and one mouthrinse product (Luvbiotics® Mouthwash with Oral Probiotics) mentioned the use of probiotics but did not specify the species. The remaining toothpaste and mouthrinse products claimed to contain probiotics and specified the probiotic species present. None of the products provided strain-level identification of the probiotics listed on their labels.

### 3.2 Probiotic culturability and species identification

Bacterial growth on MRS, blood, and LB agar plates was assessed (Table 3). Colonies grown on blood agar were considered potential contaminants, whereas colonies grown on MRS agar were considered potential probiotic isolates and were subsequently subjected to further investigation by Sanger sequencing. Sanger sequencing of these isolates revealed that only two toothpastes (Pro Bident® New Generation Toothpaste with Active Probiotics and STIM® Probiotic Toothpaste) were confirmed to contain the probiotic species *Bacillus coagulans*, consistent with their product labels. Pro Bident® New Generation Toothpaste with Active Probiotics showed moderate viability with a CFU count of log_10_ 5.41, while STIM® Probiotic Toothpaste demonstrated high viability with a CFU count of log_10_ 8.24 (Table 3).

### 3.3 Live-and total bacterial load

qPCR analysis demonstrated that among the quantifiable toothpaste products, mean total bacterial counts ranged from 4.78 log_10_ cells/mL for TP04 to 9.04 log_10_ cells/mL for TP06, while mean PMA-treated bacterial counts ranged from 4.84 log_10_ cells/mL for TP04 to 8.60 log_10_ cells/mL for TP02 (Figure 1A). Bacterial DNA detected in Henry Blooms® Probiotic Toothpaste (TP07) was below the reliable detection limit of the assay, indicating that bacterial content could not be quantified. The v-qPCR results were only consistent with CFU data for Pro bident® New Generation Toothpaste with Active Probiotics (TP05) and STIM® Probiotic Toothpaste (TP06).

**Figure 1:**
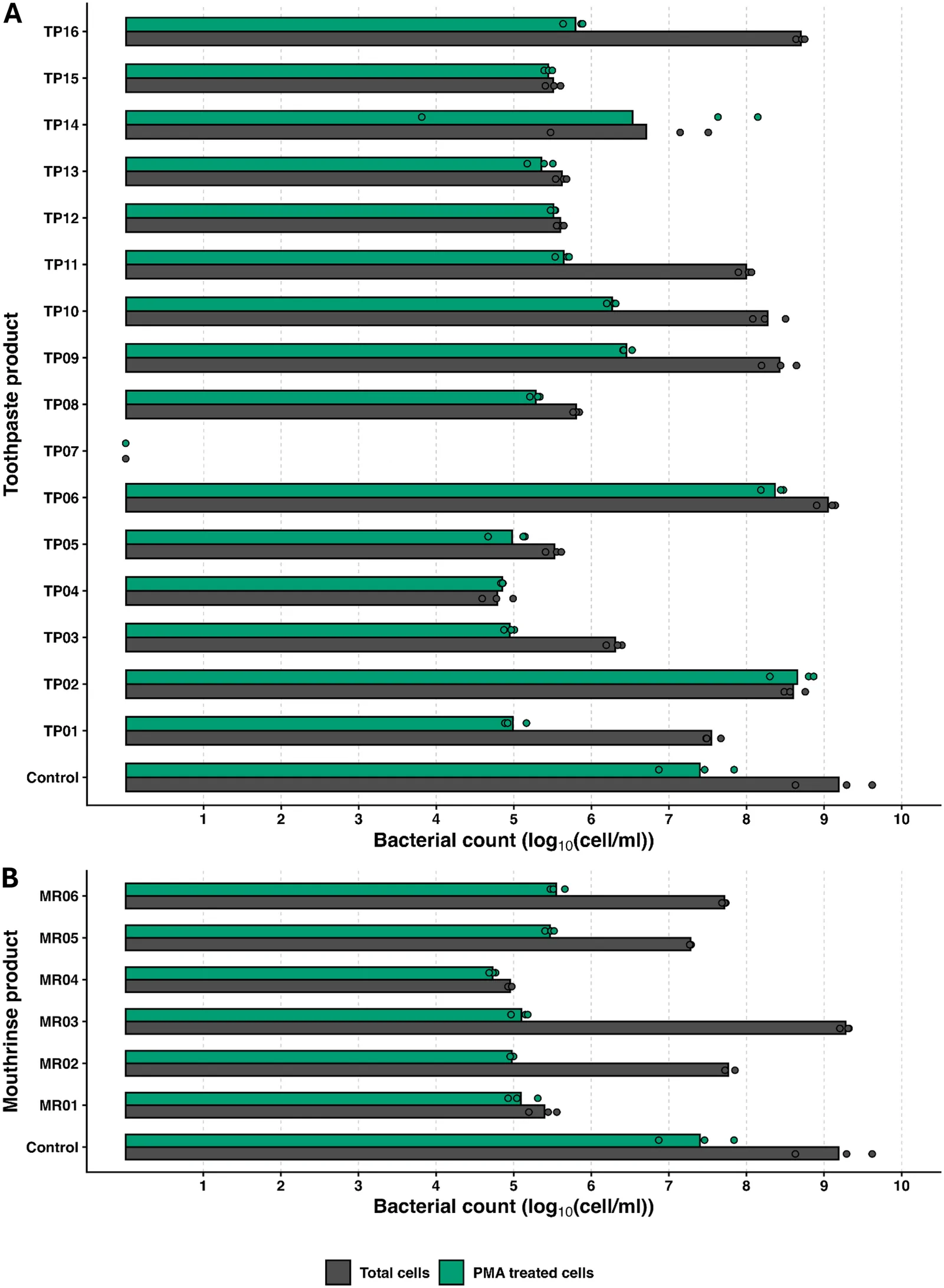
Live and total amount of bacteria of each product. Toothpastes (**A**; see Table 1) and mouthrinses (**B**; see Table 2) were analysed using viability qPCR (live cell counts) and regular qPCR (total cell counts). Individual dots represent each independent measurement, while bars represent the mean of the measurements. Data are presented as the base ten logarithm of the number of cells recovered from the products from replicates on separate days (*n* = 3 biological replicates).

For the mouthrinse products, mean total bacterial counts ranged from 4.95 log_10_ cells/mL for MR04 to 9.27 log_10_ cells/mL for MR03, while mean PMA treated bacterial counts ranged from 4.72 log_10_ cells/mL for MR04 to 5.54 log_10_ cells/mL for MR06 (Figure 1B).

### 3.4 Identification of bacterial species and potential contaminants

16S rRNA sequencing analysis revealed that TP03, TP08, and TP13 contained bacterial sequences corresponding to claimed probiotic species at low relative abundance (<10% of total detected bacterial sequences); however, the identified species did not match the probiotic species declared on the product labels. No bacterial sequences corresponding to the claimed probiotic species were detected in TP11 and TP12. The remaining 11 of 16 toothpaste samples showed detectable sequences corresponding to species listed on their product labels (Figure 2A).

**Figure 2:**
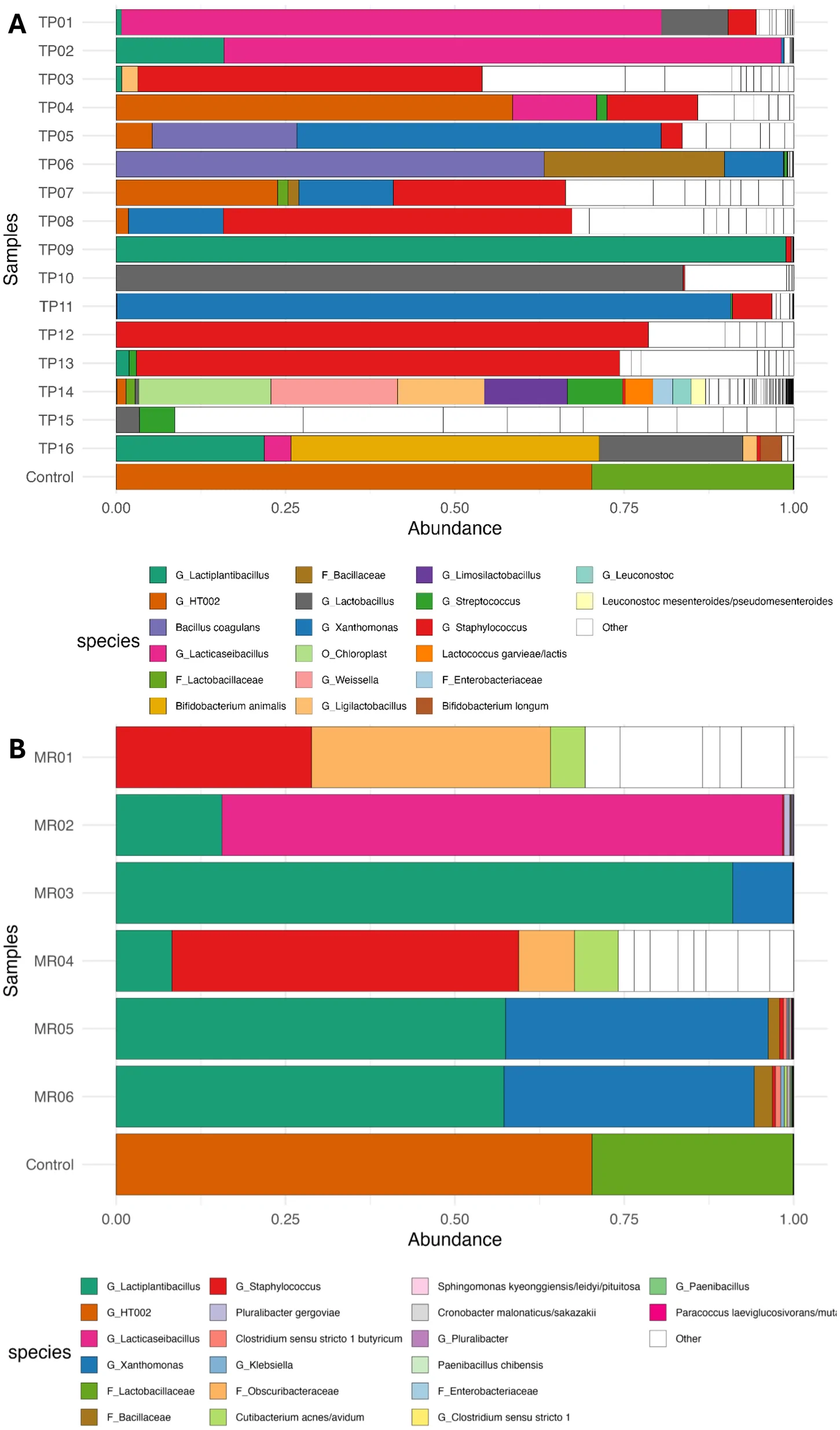
Bacterial composition in toothpaste and mouthrinse products as determined by 16S rRNA sequencing. **(A)** Relative abundance of genera (top 20) in toothpastes (Table 1). **(B)** Relative abundance of genera (top 20) in mouthrinses (Table 2). Genera at <0.1% abundance are indicated as “Other” for clarity.

Among the mouthrinse samples, no probiotic-associated bacterial sequences were detected in MR01. MR04 showed low relative abundance (<20%) of probiotic-associated bacterial sequences; however, the identified species did not correspond with the probiotic claims stated on the product label. In MR02, MR05, and MR06, bacterial species corresponding to those declared on the product labels were detected among the sequences (Figure 2B).

### 3.5 The effect of culturable probiotic products in an *in vitro* oral biofilm model

An *in vitro* multispecies biofilm model was employed to evaluate the effect of two selected probiotic-containing toothpastes (TP05 and TP06), which demonstrated high viability, culturability, and probiotic load among all tested products. Brushing the biofilms with toothpaste slurries prepared from these products did not result in a significant reduction in total biofilm. Furthermore, the overall microbial composition of the biofilms (relative abundance of commensal or pathogenic species) remained unchanged compared to the PBS control (Figures 3A,B).

**Figure 3:**
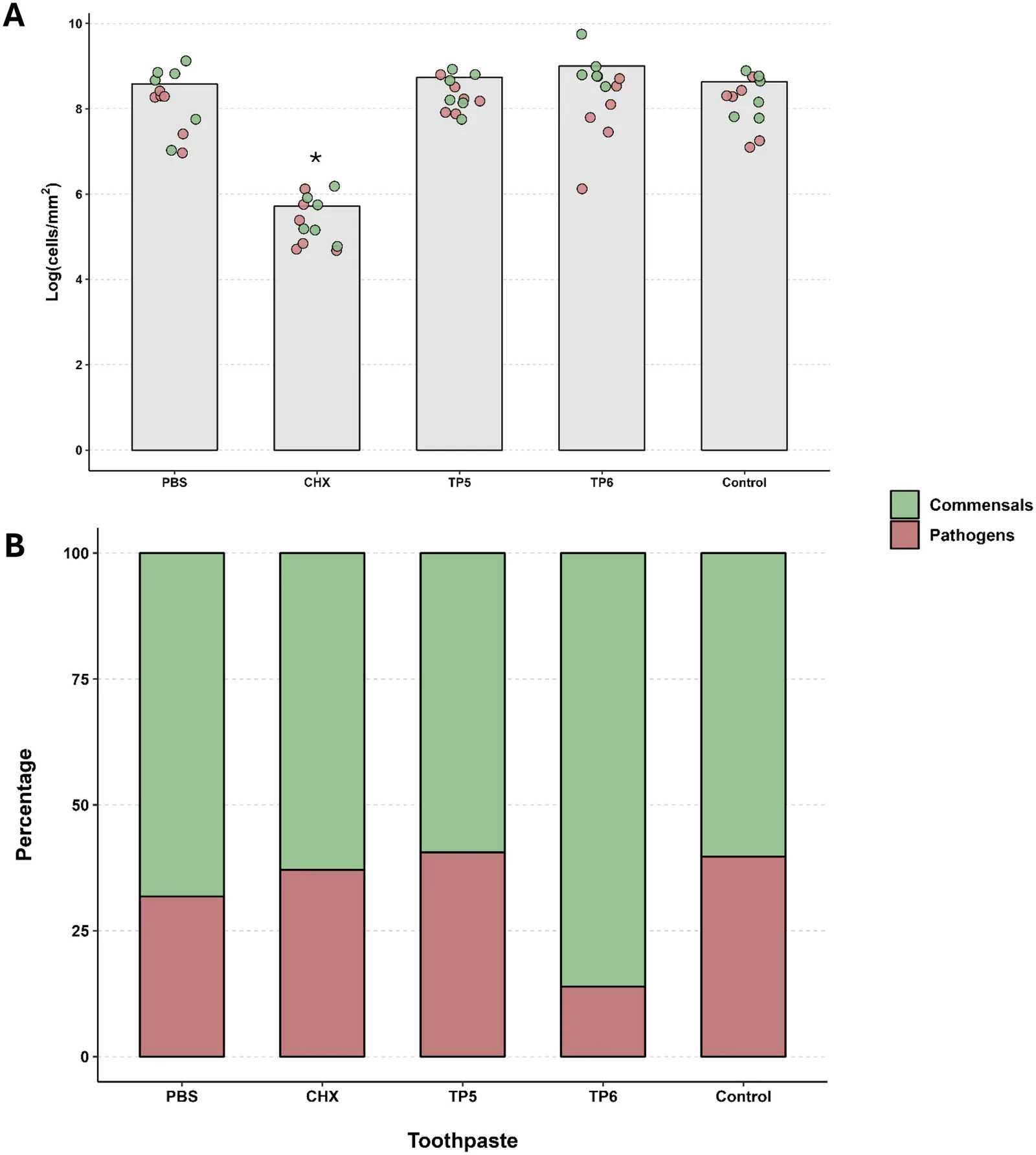
Effects of selected toothpastes (TP 5 & 6) on the *in vitro* biofilm model. **(A)** Data are expressed as total bacterial counts (log cells/mm^2^) after treatment with each product. Red dots represent the summed pathogen count in each individual replicate, while green dots represent the summed commensal count in each individual replicate. Bars represent the mean total bacterial count of the biofilm across replicates. **(B)** The ecological composition of the resulting biofilm is expressed as the mean relative abundance (%) of pathogens and commensals within the total biofilm across replicates. Significant differences between commensals and pathogens in comparison to PBS control are represented with an asterisk (two-sample *t*-test with Bonferroni correction to account for multiple comparisons, *p* < 0.05, *n* = 6 biological replicates).

### 3.6 Assessment of inhibitory activity of isolated *Bacillus coagulans* probiotic against selected oral pathogens

Of all the products, TP06 contained the most viable and accurately identified probiotic, namely *B. coagulans*. Given the limited evidence supporting B. coagulans as an oral probiotic, its potential inhibitory effect against key oral pathogens was investigated. Co-culture experiments were conducted, and bacterial viability was assessed after 6 h of incubation by comparing log-transformed values of bacterial loads in mono-cultures vs. co-cultures (Figure 4A). *B. coagulans* did not exhibit a significant inhibitory effect on the growth of the tested pathogens. Notably, *A. actinomycetemcomitans* and *S. sobrinus* demonstrated significantly increased growth in co-culture conditions; *S. mutans* showed a slight reduction in growth, although this difference was not statistically significant (Figure 4A).

**Figure 4:**
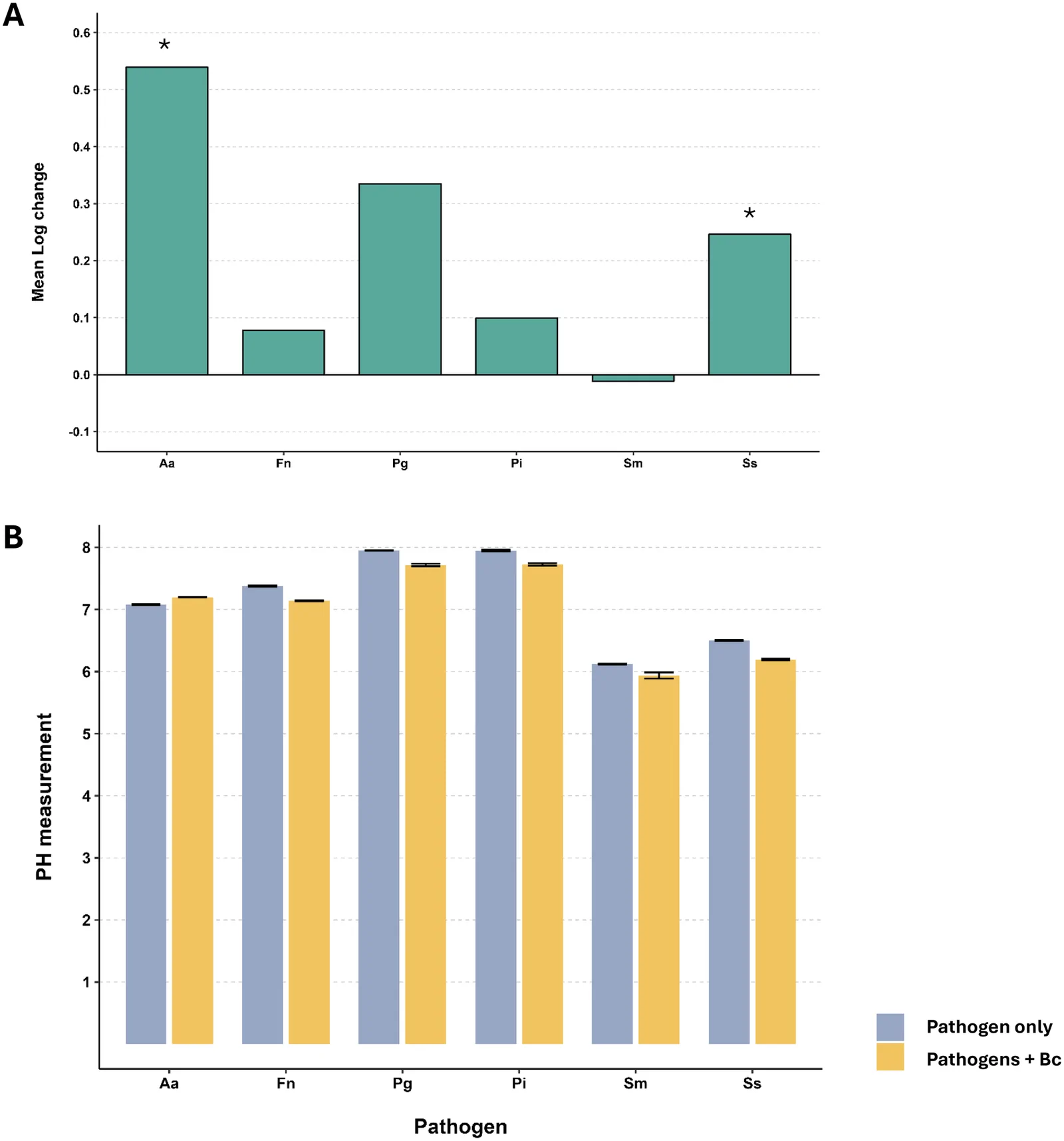
The effect of *Bacillus coagulans* (Bc) on the viability of oral pathogens. **(A)** Log change in the viability of oral pathogens after 6 h of incubation only pathogens or pathogens co-cultured with *B. coagulans*. **(B)** pH measurement of the supernatant after centrifugation of cultures containing only pathogens or pathogens co-cultured with *B. coagulans*. Bars represent the mean values across replicates. The * represents significant differences between only pathogens or pathogens co-cultured with *B. coagulans* (paired *t*-test, *p* < 0.05, *n* = 3 biological replicates).

Additionally, pH measurements of the culture supernatants post-incubation showed no substantial pH changes in the co-culture conditions, indicating that *B. coagulans* did not alter the environmental acidity to a level that could affect pathogen viability (Figure 4B).

### 3.7 Investigation of the potential postbiotic effect of *Bacillus coagulans*

To investigate whether *B. coagulans* exerts a postbiotic effect (19), a double-staining FCM assay was employed. The bacterial cell-free supernatant (CFS), considered a potential postbiotic preparation, was used to assess its impact on the viability of oral pathobionts (Figure 5).

**Figure 5:**
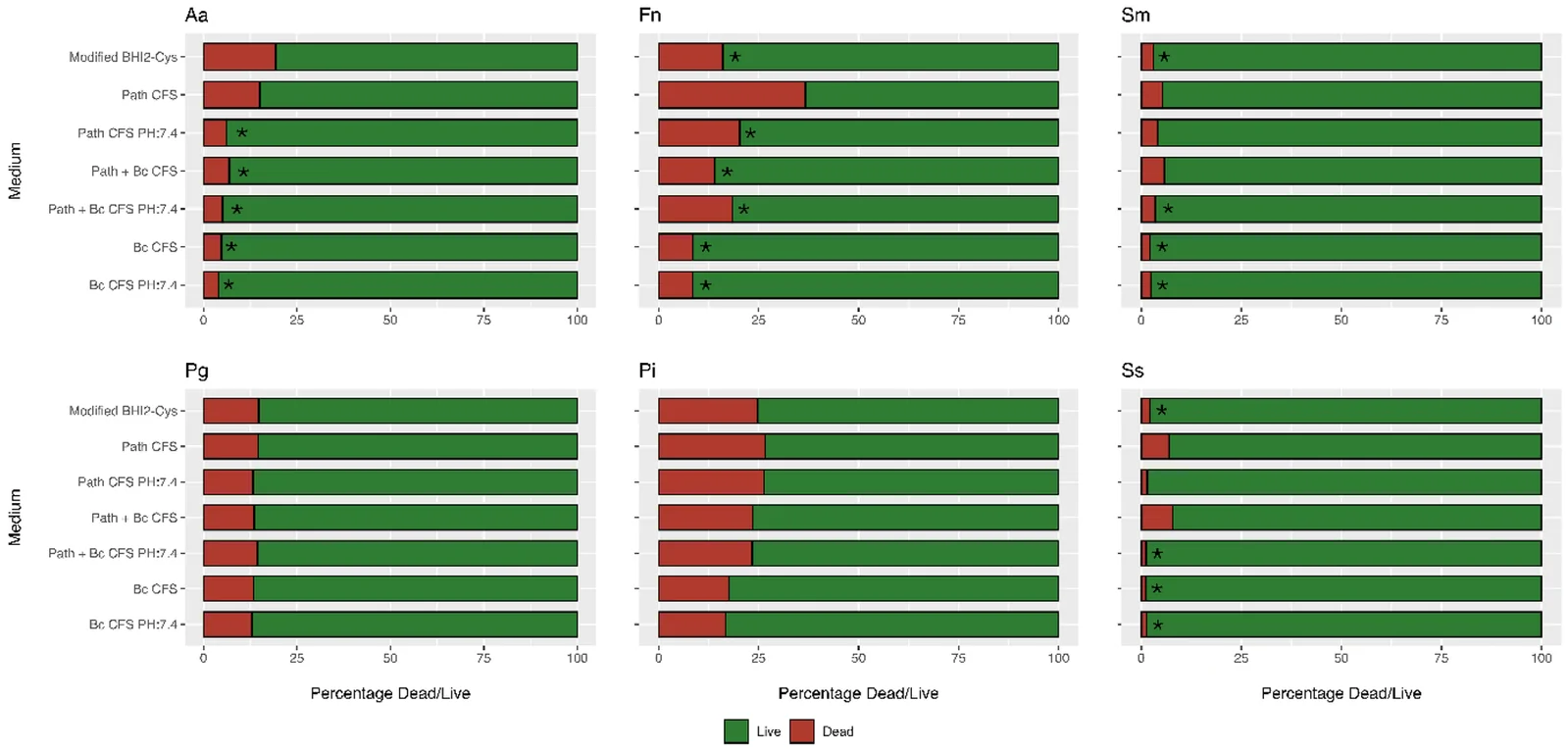
Percentage of live and dead pathogens measured by flow cytometry (FACS) after 6 h incubation in different media under aerobic or anaerobic conditions. Bars represent the mean relative abundance of pathogens and commensals across biological replicates. The * represents significant differences between Path CFS as control for other conditions (two-sample *t*-test with Bonferroni correction, *p* < 0.05, *n* = 3 biological replicates).

The pathogen-only supernatant was considered as a control (Path medium) for comparison. *B. coagulans* CFS did not result in a significant increase in dead (membrane-compromised) cells across the tested pathogens. Specifically, *P. gingivalis* and *P. intermedia* did not show any significant reduction in viable cell populations. For *F. nucleatum*, most test conditions resulted in a lower proportion of dead cells compared to the control. Similarly, *A. actinomycetemcomitans* exhibited fewer dead cells under all conditions, except in the modified BHI2-Cys medium. In S. *mutans and S. sobrinus*, decreased levels of dead cells were observed in multiple conditions, including modified BHI2-Cys, *B. coagulans* supernatant (with and without pH adjustment), and co-culture medium supplemented with *B. coagulans* at adjusted pH. These findings suggest that the *B. coagulans* CFS does not exert antibacterial or membrane-disrupting activity against the tested oral pathogens.

## 4 Discussion

Several oral hygiene products were designed with antimicrobial properties that influenced the oral microbiome. However, overconsumption can lead to the development of resistance against these antimicrobials (30, 31). In contrast, probiotics were explored as a pro-microbial approach to restore the oral microbiome balance toward a symbiotic state (32, 33). Despite the availability of probiotic oral care products, uncertainties remained regarding their actual contents. This study assessed probiotic presence, culturability, label accuracy, and effectiveness in commercially available proclaimed probiotic toothpastes and mouth rinses *(in vitro)*.

Of the 22 tested products, 20 did not contain the viable and culturable probiotic species as described on their labels. Additionally, 8 of the 22 formulations included ferments or inactivated probiotics, which do not meet the established definition of probiotics of “live microorganisms”. Moreover, all 22 products lacked specific information regarding the strain identity, which is crucial for determining strain-specific clinical efficacy, particularly in the context of oral health. In addition, several products reported bacterial names that no longer correspond to current taxonomic classification. The genus *Lactobacillus* underwent major taxonomic revision in 2020, with many species reassigned to newly established genera (34). Similarly, *Bacillus coagulans* has subsequently been reassigned to *Heyndrickxia coagulans* (35, 36). In the present study, the nomenclature stated on the product labels was retained to ensure consistency throughout the dataset.

Among the 22 selected probiotic-containing toothpastes and mouthrinses, only two showed visible colony growth on MRS agar plates. Moreover, Sanger sequencing revealed that the bacterial species recovered from both products, Pro Bident® New Generation Toothpaste with Active Probiotics and STIM® Probiotic Toothpaste, matched the species declared on their labels. Both products contained *Bacillus* species, but only STIM Probiotic Toothpaste accurately listed *Bacillus coagulans* on the label. Due to its ability to form spores, *Bacillus coagulans* is suitable for use in water-based formulations, as the spores can survive manufacturing conditions and remain viable in such environments (37, 38).

To distinguish between live and dead cells, PMA treatment was applied before qPCR (39). PMA intercalates with free DNA from membrane-compromised cells, preventing its amplification, thereby enriching the signal from viable cells. Although v-qPCR is a well-established method for viability assessment, its efficacy can be influenced by sample composition and the presence of contaminants. Toothpaste and mouthrinse formulations may contain ingredients that may interfere with PMA activity by either inhibiting intercalation or altering cell membrane permeability (40, 41). Additionally, the presence of viable but non-culturable (VBNC) bacteria may further complicate interpretation, as these cells can evade both culture-based detection and PMA exclusion, yet still retain metabolic activity and be detected as viable cells in qPCR analyses (21, 42). This discrepancy can also partly be explained by the fact that qPCR detects DNA fragments regardless of cellular viability. Even after fermentation or pasteurization, non-viable bacteria or bacterial remnants may retain intact DNA segments long enough to be amplified by qPCR (43, 44).

The bacterial species identified by sequencing in the samples did not consistently correspond to those commonly associated with proven oral health benefits, such as *Limosilactobacillus reuteri* or *Streptococcus salivarius* strains (32, 45). This raises concerns regarding the actual functional value of these products in supporting oral health.

CFU, qPCR, and sequencing analysis confirmed that only two toothpaste products (Pro Bident® New Generation Toothpaste with Active Probiotics and STIM® Probiotic Toothpaste) contained a consistent and viable amount of *Bacillus coagulans* across replicates. These two products were further tested for their *in vitro* effect on oral biofilms. However, no reduction of pathogenic bacteria or significant decrease in total bacterial load in the multi-species biofilm was observed in test groups, suggesting that these products did not exert a measurable antimicrobial effect under the tested *in vitro* conditions.

Aside from the two products, *B. coagulans* was isolated, and its potential inhibitory effects were evaluated on six oral pathogens through co-culturing, as well as potential postbiotic activity by exposing the pathogens to its cell-free supernatant. No inhibitory effect of *B. coagulans* was observed under the tested conditions. This aligns with the uncertainties reported in the literature regarding the effectiveness of *B. coagulans* in oral health applications (46). However, the use of nutrient-rich BHI medium should be considered when interpreting these findings, as such conditions may promote microbial growth and do not fully reflect the nutrient conditions encountered in saliva (47, 48).

In comparison with the previous study by Van Holm et al. on probiotic tablets, the current findings in probiotic-containing toothpastes and mouthrinses reveal several similar concerns regarding product quality and probiotic authenticity (22). In both studies, many products failed to contain the declared viable probiotic strains. Furthermore, incomplete strain-level information and the inclusion of non-viable bacterial components were identified as recurring issues, raising concerns regarding compliance with the accepted definition of probiotics as live microorganisms. However, formulation characteristics may partly explain some differences between the product categories. While tablet formulations generally provide a relatively dry environment that may better preserve bacterial stability during storage, toothpastes and mouthrinses contain water, preservatives, and other active ingredients that may adversely affect bacterial survival and culturability. These formulation-dependent factors may contribute to the limited recovery of viable bacteria observed in the current study. Taken together, the findings from both studies suggest that the challenges related to viability, strain identification, and labeling accuracy are not restricted to a specific product but rather represent a broader issue within commercially available oral probiotic products (22).

A further limitation of the present study is that only one independent lot of each commercial product was analysed. Therefore, the findings should be interpreted as specific to the batches tested, as product composition, microbial viability, and manufacturing consistency may vary between batches. Future studies should include multiple independent lots of the same products to assess batch-to-batch variability and improve the generalisability of the findings.

Despite the limitations, the consistency and accuracy of our control samples across all three replicates confirm the reliability of both qPCR and sequencing pipelines in our experimental setup. These controls consistently yielded correct and repeatable results, supporting the overall validity of the analytical methods employed.

Future studies should evaluate a larger and more diverse range of probiotic toothpastes and mouthrinses, including independent batches of the same products and a greater number of experimental replicates. Additional *ex vivo* and *in vivo* investigations are also needed to determine whether these formulations can effectively deliver and retain probiotic microorganisms within the oral cavity.

## 5 Conclusion

This study demonstrated that, under the experimental conditions used, none of the commercially available mouthrinses tested contained culturable probiotic species, and only two out of 16 toothpaste products yielded culturable bacteria consistent with their labels. Even among these two products, the identified species (*B. coagulans*) did not exhibit inhibitory activity against common oral pathogens.

Compared with probiotic lozenges, which are commonly used as an established delivery system for oral probiotics, the currently available probiotic toothpaste and mouthrinse products may not consistently meet the established criteria for probiotics. This highlights the need to critically reassess the use of probiotic-containing toothpastes and mouthrinses as reliable probiotic delivery systems, particularly in scientific research, while also emphasizing the need for more accurate use of the term “probiotic” in product marketing and commercial claims. These findings apply to the specific product batches analysed and should not be considered a definitive assessment of each commercial product or brand as a whole.

## Data Availability

The raw data supporting the conclusions of this article will be made available by the authors, without undue reservation.
