# Supplementary material for: Probiotic toothpastes and mouthrinses: probiotic presence, viability, culturability, label accuracy, and effectiveness in vitro
Source: Front Oral Health. 2026 Oct 15;7:1936217. doi: 10.3389/froh.2026.1936217 (PMC13716578; doi:10.3389/froh.2026.1936217)
Supplement: Supplementary file 1 [file Datasheet1.pdf]

**Supplemental Table1:** Used primers and probes

| Species | | Primers & Probe (Final concentrations) | Amplicon length (bp) | Target |
|---|---|---|---|---|
| *Aggregatibacter actinomycetemcomitans* | Forward | CGG TGT CGA TTT GGG GAT TGG (300 nM) | 237 | 16S rRNA gene |
| | Reverse | TGC AGC ACC TGT CTC AAA GC (300 nM) | | |
| | Probe | AGA ACT CAG AGA TGG GTT TGT GCC TTA GGG (100 nM) | | |
| *Prevotella intermedia* | Forward | TGT GCC CYT TTG CAT TTA CCC TTC (300 nM) | 216 | 16S rRNA gene |
| | Reverse | CAC CAT GAA TTC CGC ATA CG (900 nM) | | |
| | Probe | TGG CGG ACT TGA GTG CAC GC (200 nM) | | |
| *Porphyromonas gingivalis* | Forward | CCG TAA GAA TAA GCA TCG GCT AAC TC (300 nM) | 195 | 16S rRNA gene |
| | Reverse | CAC GAA TTC CGC CTG C (300 nM) | | |
| | Probe | CAC TGA ACT CAA GCC CGG CAG TTT CAA (100 nM) | | |
| *Fusobacterium nucleatum* | Forward | GGA TTT ATT GGG CGT AAA GC (300 nM) | 191 | 16S rRNA gene |
| | Reverse | ATC TGT CCA GTA AGC TGG CTT CC (300 nM) | | |
| | Probe | CTC TAC ACT TGT AGT TCC G (300 nM) | | |
| *Streptococcus mutans* | Forward | GCC TAC AGC TCA GAG ATG CTA TTC T (900 nM) | 114 | gtfB gene |
| | Reverse | GCC ATA CAC CAC TCA TGA ATT GA (900 nM) | | |
| | Probe | TGG AAA TGA CGG TCG CCG TTA TGA A (100 nM) | | |
| *Streptococcus sobrinus* | Forward | AAA TAC GGC CAG TGC CAA AG (200 nM) | 165 | gtfT gene |
| | Reverse | CCA GCC TGA GAT TCA GCT TGT (200 nM) | | |
| | Probe | CCT GCT CCA GCG ACA AAG GCA GC (250 nM) | | |
| *Actinomyces naeslundii* | Forward | TCG AAA CTC AGC AAG TAG CCG (200 nM) | 96 | gene encoding an unknown protein |
| | Reverse | AGA GGA GGG CCA CAA AAG AAA (200 nM) | | |
| | Probe | GGG TAC TCT AGT CCA AAC TGG CGG ATA GCG (100 nM) | | |
| *Actinomyces viscosus* | Forward | GTG AAG GAG CCA GCT TGC TGG TTC TG (200 nM) | 155 | 16S rRNA gene |
| | Reverse | CGG AAC AAA CCT TTC CCA GGC (200 nM) | | |
| | Probe | ATG AGT GGC GAA CGG GTG AGT AAC (125 nM) | | |
| *Veillonella parvula* | Forward | GAC GAA AGT CTG ACG GAG CA (200 nM) | 171 | 16S rRNA gene |
| | Reverse | TGC CAC CTA CGT ATT ACC GC (200 nM) | | |
| | Probe | AGC TCT GTT AAT CGG GAC GAA AGG C (125 nM) | | |
| *Streptococcus oralis* | Forward | ACC AGC AGA TAC GAA AGA AGC AT (400 nM) | 229 | *gtfR* gene |
| | Reverse | AGG TTC GGG CAA GCG ATC TTT CT (400 nM) | | |
| | Probe | AAG GCT GCT GTT GCT GAA GAA GT (100 nM) | | |
| *Streptococcus sanguinis* | Forward | CAA AAT TGT TGC AAA TCC AAA GG (600 nM) | 75 | *gtfP* gene |
| | Reverse | GCT ATC GCT CCC TGT CTT TGA (600 nM) | | |
| | Probe | AAA GAA AGA TCG CTT GCC AGA ACC GG (100 nM) | | |
| *Streptococcus gordonii* | Forward | GAA GAA CTG GGT AGC GAT TGC T (400 nM) | 262 | *gtfG* gene |
| | Reverse | GTT AGC TGT TGG ATT GGT TGC C (400 nM) | | |
| | Probe | AGA ACA GTC CGC TGT TCA GAG CAA (100 nM) | | |
| *Streptococcus mitis* | Forward | GGC TCG TAG TCT GGA GAT GG (600 nM) | 133 | 16S rRNA gene |
| | Reverse | TAG GTC GTC GTC CCA AGG AA (600 nM) | | |
| | Probe | CGA AGA GCA CCA ATA GCA CCT CCC (140 nM) | | |
| *Streptococcus salivarius* | Forward | GAC GAT GAC TGT CAA CTT GAC AC (400 nM) | 247 | *Dextranase* gene |
| | Reverse | ACC GTA ACG TGG GAA AAC TG (400 nM) | | |
| | Probe | GTA GCG TCA GAG TGG TTG AC (100 nM) | | |
| *L. reuteri* DSM17938 | Forward | TTAAGGATGCAAACCCGAAC (300 nM) | 177 | gene encoding an unknown protein |
| | Reverse | CCTTGTCACCTGGAACCACT (300 nM) | | |
| | Probe | FAM- ATGACCTTGTAGGAGCCGTCTGGATAC -Tamra (100 nM) | | |
| *L. reuteri* ATCC PTA5289 | Forward | GACAGTGGCTAAACGCCTTC (300 nM | 200 | gene encoding an unknown protein |
| | Reverse | AATTCCACTTGCCATCTTCG (300 nM) | | |
| | Probe | FAM-TTGGTAGGCTTGTTGAAAATCGCTA-TARMA (100 nM) | | |

**Supplemental Table 2:** Specifications of the evaluated products, including product name, country of origin, expiry date, recommended storage conditions, and batch or lot number. (All products were stored according to the manufacturers' indicated storage conditions and were tested within their stated expiry dates.)

| Nr | Product Name | Country | Expiry date | Storage conditions | Batch/Lot number |
|---|---|---|---|---|---|
| TP 01 | Gutology® Probiotic Toothpaste (120ml) | United Kingdom | 12 month after opening | NR | 24171 |
| TP 02 | Hyperbiotics® Probiotic Toothpaste with Activated Charcoal | United States | NR | NR | DT82MFG0722 |
| TP 03 | Luvbiotics® original toothpaste with probiotics | United Kingdom | 6 month after opening | 5–25 °C | LOT23283 |
| TP 04 | YUUU® advance probiotic and pine needle toothpaste | Korea | 10/5/2026 | NR | JO1 |
| TP 05 | Pro bident® New Generation Toothpaste with Active Probiotics | Turkey | 6 month after opening | 5–30 °C | 9830525 |
| TP 06 | STIM® Probiotic Toothpaste (80 G) | India | Oct-25 | Cool, dry, dark place, away from direct sunlight | HHB104 |
| TP 07 | Henry blooms® probiotic toothpaste | Australia | Aug-26 | Room temperature, away from heat and direct sunlight | 20291 |
| TP 08 | REACH Microbiome® Whitening Toothpaste, Natural Probiotic Ingredients | Korea | 9/8/2025 | NR | CH1UA |
| TP 09 | OraLactin® ApaCare Pre- and Postbiotic Probiotic Toothpaste | Germany | 12 month after opening | NR | 400908 |
| TP 10 | Biorepair®, Peribiome Gum Protection Toothpaste, with probiotics | Italy | 12 month after opening | NR | 332051045 |
| TP 11 | Gaia® natural probiotic toothpaste | Australia | NR | below 25 °C | (B)2E01 |
| TP 12 | NANB® + (enzyme whitening balancing toothpaste) | China | 10/11/2026 | Cool, dry, away from direct sunlight | 957205 |
| TP 13 | Sp-4® Toothpaste Probiotic Whitening Toothpaste Yayashi | China | 3 Years | NR | 7/6/2042 |
| TP 14 | ELAA®.Lava Toothpaste \| Probiotic Whitening Charcoal Mint | NR | NR | NR | NR |
| TP 15 | Dentist Plus® White Premium Toothpaste Xylitol and Probiotics | Thaniland | Nov-28 | NR | 3003211123 |
| TP 16 | Be Well® Natural Toothpaste with probiotics | NR | NR | NR | NR |
| MR 01 | Luvbiotics® Mouthwash with Oral Probiotics | United Kingdom | 6 month after opening | 5–25 °C | LOT5487 |
| MR 02 | Riven® rinse probiotic mouthwash | United States | NR | NR | X0033HVIYZ |
| MR 03 | OraLactin® Pre and Postbiotic Mouthwash | Germany | 12 month after opening | NR | LOT322021 |
| MR 04 | Henry blooms® on the go probiotic mouthwash | Australia | Dec-26 | Room temperature, away from heat and direct sunlight | LOT211221A |
| MR 05 | Truthpaste® mint probiotic mouthwash fluoride free | United Kingdom | NR | 5–25 °C, away from direct sunlight | NR |
| MR 06 | Truthpaste® mint probiotic mouthwash with fluoride | United Kingdom | 30/08/25 | 5–25 °C, away from direct sunlight | 3008242 |

* NR: Not Reported

## Supplementary Data

**P-values for Figure 3A (pathogens):**

| | CHX | Cont | PBS | TP5 |
|---|---|---|---|---|
| Cont | 0.032 | - | - | - |
| PBS | 0.020 | 1.000 | - | - |
| TP5 | 0.020 | 1.000 | 1.000 | - |
| TP6 | 0.032 | 1.000 | 1.000 | 1.000 |

**P-values for Figure 3A (commensals):**

| | CHX | Cont | PBS | TP5 |
|---|---|---|---|---|
| Cont | 0.048 | - | - | - |
| PBS | 0.048 | 1.000 | - | - |
| TP5 | 0.040 | 1.000 | 1.000 | - |
| TP6 | 0.040 | 0.324 | 0.792 | 0.370 |

**P-values for Figure 3B (% pathogens and commensals):**

| | CHX | Cont | PBS | TP5 |
|---|---|---|---|---|
| Cont | 1.00 | - | - | - |
| PBS | 1.00 | 1.00 | - | - |
| TP5 | 1.00 | 1.00 | 1.00 | - |
| TP6 | 0.36 | 0.98 | 1.00 | 0.38 |

**P-values for Figure 4A:**

| Path | p-value |
|---|---|
| Aa | 0.000414 |
| Fn | 0.289732 |
| Pg | 0.186326 |
| Pi | 0.339818 |
| Sm | 0.929297 |
| Ss | 0.001632 |

**P-values for Figure 4B:**

| Path | p-value |
|---|---|
| Aa | 0.115 |
| Fn | 0.095 |
| Pg | 0.145 |
| Pi | 0.074 |
| Sm | 0.087 |
| Ss | 0.128 |

**P-values for Figure 5:**

**Aa**

| Condition compared with Path medium | p-value |
|---|---|
| Bc CFS | 0.0022895 |
| Bc CFS pH 7.4 | 0.0012158 |
| Modified BHI2-Cys | 0.4156044 |
| Path + Bc CFS | 0.0159363 |
| Path + Bc CFS pH 7.4 | 0.0031605 |
| Path medium pH 7.4 | 0.0079621 |

**Fn**

| Condition compared with Path medium | p-value |
|---|---|
| Bc CFS | 0.0000000 |
| Bc CFS pH 7.4 | 0.0000000 |
| Modified BHI2-Cys | 0.0000006 |
| Path + Bc CFS | 0.0000002 |
| Path + Bc CFS pH 7.4 | 0.0000030 |
| Path medium pH 7.4 | 0.0000106 |

**Sm**

| Condition compared with Path medium | p-value |
|---|---|
| Bc CFS | 0.0002266 |
| Bc CFS pH 7.4 | 0.0004211 |
| Modified BHI2-Cys | 0.0036817 |
| Path + Bc CFS | 0.9113672 |
| Path + Bc CFS pH 7.4 | 0.0283800 |
| Path medium pH 7.4 | 0.2311563 |

**Ss**

| Condition compared with Path medium | p-value |
|---|---|
| Bc CFS | 0.0002266 |
| Bc CFS pH 7.4 | 0.0004211 |
| Modified BHI2-Cys | 0.0036817 |
| Path + Bc CFS | 0.9113672 |
| Path + Bc CFS pH 7.4 | 0.0283800 |
| Path medium pH 7.4 | 0.2311563 |

**Pg**

No significant differences were observed among the different media conditions ($p = 0.89$)

**Pi**

No significant differences were observed among the different media conditions ($p = 0.89$)
